# Bi-Directional Mutual Energy Trade between Smart Grid and Energy Districts Using Renewable Energy Credits

**DOI:** 10.3390/s21093088

**Published:** 2021-04-29

**Authors:** Sana Rehman, Bilal Khan, Jawad Arif, Zahid Ullah, Abdullah J. Aljuhani, Ahmad Alhindi, Sahibzada M. Ali

**Affiliations:** 1 Department of Electrical & Computer Engineering, Abbottabad Campus, COMSATS University Islamabad, Abbottabad 22060, Pakistan; sana.rehman@outlook.com (S.R.); bilalkhan@cuiatd.edu.pk (B.K.); hallianali@cuiatd.edu.pk (S.M.A.); 2 School of Electrical Engineering & Computer Science (SEECS), National University of Sciences & Technology (NUST), Islamabad 44000, Pakistan; jawad.arif@seecs.nust.edu.pk; 3 Department of Electrical Engineering, Sialkot Campus, University of Management and Technology Lahore, Sialkot 51310, Pakistan; 4 Department of Electrical and Computer Engineering, King Abdulaziz University, Jeddah 21589, Saudi Arabia; ajaljohani@kau.edu.sa; 5 Center of Excellence in Intelligent Engineering Systems, King Abdulaziz University, Jeddah 21589, Saudi Arabia; 6 Department of Computer Science, Umm Al-Qura University, Makkah 24381, Saudi Arabia; ahhindi@uqu.edu.sa

**Keywords:** blockchain, peer-to-peer, Solar Coin, energy transactions, energy districts, central authority

## Abstract

A central authority, in a conventional centralized energy trading market, superintends energy and financial transactions. The central authority manages and controls transparent energy trading between producer and consumer, imposes a penalty in case of contract violation, and disburses numerous rewards. However, the management and control through the third party pose a significant threat to the security and privacy of consumers’/producers’ (participants) profiles. The energy transactions between participants involving central authority utilize users’ time, money, and impose a computational burden over the central controlling authority. The Blockchain-based decentralized energy transaction concept, bypassing the central authority, is proposed in Smart Grid (SG) by researchers. Blockchain technology braces the concept of Peer-to-Peer (P2P) energy transactions. This work encompasses the SolarCoin-based digital currency blockchain model for SG incorporating RE. Energy transactions from Prosumer (P) to Prosumer, Energy District to Energy District, and Energy District to SG are thoroughly investigated and analyzed in this work. A robust demand-side optimized model is proposed using Genetic Algorithm (GA) and Particle Swarm Optimization (PSO) to maximize Prosumer Energy Surplus (PES), Grid revenue (GR), percentage energy transactions accomplished, and decreased Prosumer Energy Cost (PEC). Real-time averaged energy data of Australia are employed, and a piece-wise energy price mechanism is implemented in this work. The graphical analysis and tabular statistics manifest the efficacy of the proposed model.

## 1. Introduction

Smart Grid (SG) has changed the way electricity is generated and consumed due to the adoption of the: (a) Advanced computational platform, (b) bi-directional communication of energy, (c) satisfying consumers need through reduced energy cost, and (d) automated, smart, secure network [1]. Energy Districts (EDs) permit small-scale Renewable Energy (RE) consumers and prosumers to trade energy locally [2], an evolving concept of environment-friendly energy transfer.

In conventional centralized energy systems, a central authority manages the energy transfer between consumers and prosumers. The central authority is responsible for supervising agreements and accomplishing transparent trading among participants [3]. However, the outlook of energy with the concept of prosumers accompanied with SG is complicated and challenging [4]. The intermittent RE resources demand for storage options to store surplus energy within SG. Therefore, the most important feature of transportation electrification is the Vehicle to Grid (V2G) concept that store the surplus energy and ensure the provision of surplus energy to SG during high load demand. The storage capability of V2G enhances the utilization of renewable energy sources within EDs [5,6]. The nonlinear and stochastic data within SG demands for advanced machine learning methods. Machine learning is an efficient tool to design energy management model for bidirectional energy and data flow between SG and ED [7]. The complex energy future with the need of integrating more and more RE in the power grid cannot be effectively managed through the conventional centralized system; thus, an alternative decentralized market approach is required.

In a traditional centralized network, more time and money of consumers are wasted involving a third party. The consumers encounter high transaction and management costs and have no direct access to the energy market [8]. The critical centralized architecture results in a computational burden on central authority and is greatly at threat of single-point failure [9]. The challenges associated with the conventional centralized energy network can be addressed through a decentralized approach surpassing the central authority. Blockchain technology is a widely emerging decentralized concept. Blockchain provides a platform for consumers and prosumers to buy and sell energy in a peer-to-peer (P2P) fashion [10] directly with each other and with the SG, bypassing the third party. P2P energy transactions reduce the management and transaction cost and permit consumers to have direct access to the energy market. Due to its decentralized nature blockchain is immune to a single-point failure.

The cost of running decentralized networks and mining cryptocurrencies requires a tremendous amount of energy resulting in an expensive decentralized blockchain network. The high cost of mining cryptocurrencies restricts the blockchain network to the moneyed community. Given the aforementioned issues incurred in decentralized blockchain networks, advance research is focusing on a cryptocurrency that assists a cost-effective network [11]. SolarCoin (SLR)-based blockchain is an evolving platform that addresses the issue of high energy consumption of another cryptocurrencies-based blockchain [12]. A cost-effective SLR blockchain network lays out an equal opportunity platform. Any person owning some SLR stakes is invited to join the blockchain network. Besides cracking the energy consumption problem, SLR encourages the transition from fossil fuel to clean green energy. Small or large-scale solar energy producers registered with Solar Coin Foundation (SCF) earns one SLR as a free incentive at the rate of one MW produced. Encouraging the users to install and rely on solar panels rather than the conventional fossil fuel energy method, through disbursing one SLR for each MWh as a free reward, is contributing towards a carbon-free environment. SLRs can be exercised for any purpose or can be converted to fiat currency [13].

Extensive research works have been dedicated to blockchain technology over the last few years [8,9,10,11,12,13,14,15,16,17,18,19,20,21,22,23]. Authors in [14] proposed IBM HyperLedger Fabric blockchain-based P2P crowdsourced energy model. Authors in [15] presented demand-side management permitting users to reduce their electricity bill through effective day-ahead scheduling of energy consumption incorporating the blockchain technology. In [16], authors explored ongoing blockchain-based microgrid projects and came with the idea of establishing a blockchain-based microgrid. Authors in [8] came up with the architecture of solar energy production and distribution incorporating Smart Contracts to establish an energy exchange market. In [3], authors proposed the design and implementation of a Decentralized Transactive platform encompassing fault detection. Authors in [17] proposed the design and implementation of local energy markets on a private blockchain and evaluation of an economic market mechanism.

A case study incorporating implemented Ethereum-based Brooklyn Microgrid (BMG) was evaluated by researchers in [2]. Authors in [18] proposed a privacy-preserving blockchain-enabled trading model. The authors addressed the privacy protection of individuals on consortium blockchain-based energy trading systems through the introduction of a noise-based privacy-preserving approach. In [19], authors evaluated blockchain-based demand–response distributed energy management for SGs. Effective distributed management through the detection of energy unbalances and disbursement of rewards and penalties was implemented using Smart Contracts. Authors in [20] proposed the implementation of blockchain technology to empower Machine-to-Machine (M2M) interactions in a chemical industry based on a proof-of-concept. The employment of Smart Contracts to enhance the speed, security, and scalability of blockchain-based energy exchange was proposed by authors in [21]. Different blockchain solutions were tested on the Pacific Northwest National Lab (PNNL)’s transactive campus to investigate and reduce possible associated cyber risks.

Although the above-mentioned research works successfully explored and implemented the characteristics and applications of blockchain technology in the energy market and SG, they lack in dispensing (a) the SLR blockchain-based Mutual Energy Trade Model (METM) incorporating demand-side management, (b) the evaluation of optimized energy transactions accomplished in a blockchain network considering seasonal variations, and (c) the implementation of energy transactions between prosumers, consumers, and multi-EDs based on SLR blockchain. Moreover, free SLRs disbursement as an incentive for Solar Energy Prosumers was not incorporated in previous works.

Significant contributions of this work based on the previous-mentioned issues are:

- SLR blockchain-based energy trade model is designed and analyzed that deploys Smart Contracts to empower mutual energy transactions between Prosumer and Consumer, Prosumer and Prosumer, multiple EDs, and between ED and SG. Three EDs are considered in this work with each ED comprising of N-consumers and N-prosumers. EDs are interfaced with SG as ED1 and ED2 are Solar Energy Districts and ED3 is Wind Energy District.
- A demand-side multi-objective optimization model is implemented. The Australian-based piece-wise real-time pricing scheme is considered. Price-based incentives are considered for prosumers to achieve the objectives of maximizing Prosumer Energy Surplus (PES), Grid Revenue (GR), percentage energy transactions accomplished, and minimization of Prosumer Energy Cost (PEC).
- Incentives coupled with SLR blockchain are considered for Solar Energy Prosumers. The performance of the proposed model is evaluated using a Genetic Algorithm and Particle Swarm Optimization considering seasonal variations. In-depth tabular and graphical comparison of GA optimized results and PSO is critically analyzed.

Table 1 summarizes the comparative analysis of selected research work conducted on blockchain technology. Table 1 also highlights the contributions of this work compared to existing research work. In Table 1, “🗸” represents the presence of the attributes and “🗴” represents the absence of the attributes.

The rest of the paper is organized as follows. Section 2 presents the proposed SLR blockchain-based METM. A blockchain-based optimization model is discussed in Section 3. Section 4 covers the performance evaluation accompanied by the data analysis, seasonal variations, blockchain implementation, and tabular statistics. The paper concludes with a summary and future directions in Section 5.

## 2. SolarCoin (SLR) Blockchain-Based Mutual Energy Trade Model (METM)

This section presents the proposed SLR Blockchain-based METM. The pricing mechanism and mutual energy contracts employed in this work are also explicated in this section.

### 2.1. System Model

**Figure 1:**
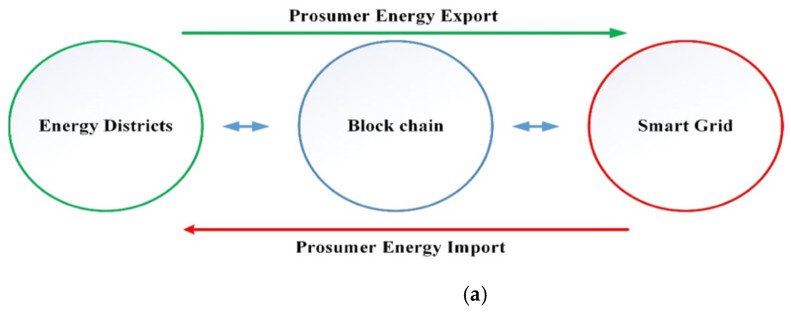

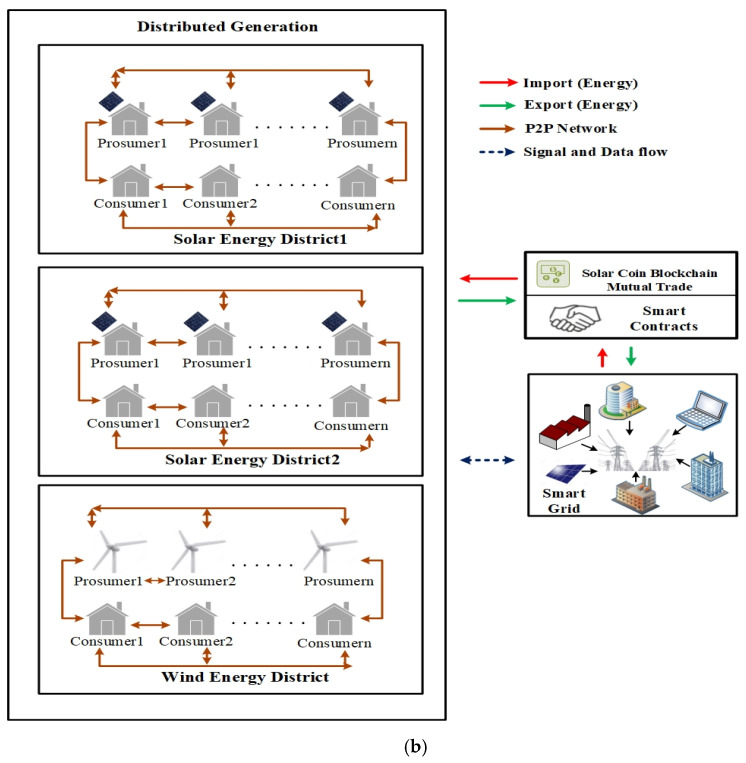
(**a**) Blockchain-based bi-directional METM, (**b**) SLR Blockchain-based bi-directional METM.

Figure 1 presents the proposed blockchain-based energy model, which comprises three distributed EDs: Two Solar EDs and a wind ED. Each Solar ED includes N-consumers and N-Solar Energy Prosumers (SEPs). Similarly, Wind ED incorporates N-Wind Energy Prosumers (WEPs) accompanied by N-consumers. The energy demand of three EDs cannot be met alone through RE and are interfaced with SG. Three EDs also support SG during peak hours. The energy transactions between multiple prosumers, consumers, EDs, and SG are carried on a P2P SLR blockchain platform, as shown in Figure 1.

Transactions executed on a digital network eliminating the third party contribute to a cost-effective system. SEPs and WEPs produce energy, satisfy their load requirements, and share the surplus, unused energy with neighboring consumers. Blockchain permits prosumers and consumers to trade energy transparently and securely without relying on the grid. Consumers and Prosumers on the network can buy energy at a reduced cost and are paid for selling excess energy to neighboring users and the grid. Participants on the blockchain network are referred to as *nodes.* SLR is exchanged among the nodes of the network as a result of energy transactions.

In Figure 1, SEPs and WEPs update their status on the network claiming to sell energy. Consumers wanting to buy energy put requests on the network.

Prosumers and consumers on the blockchain network undertake an agreement under the umbrella of a contract without the need of trusting each other. The nodes must fulfill contract conditions to carry out energy transactions. The contract will be executed automatically after the conditions are satisfied. The system (a) connects prosumer and consumer on the network and scrutinizes (b) energy availability according to consumer’s request, (c) Consumer’s balance, and SLRs at hand. On satisfactory execution of the contract, the transaction record is dispensed to each node of the network for verification. After verification, the transaction is accomplished, and a block of the transaction is added to the chain of the network.

### 2.2. Pricing Mechanism

The Queensland, Australia-based piece-wise energy price mechanism is implemented in this work. In this pricing mechanism, consumers are charged according to (a) the amount of energy utilized and (b) the day and time of energy utilization. Consumers encounter high energy utilization rates during peak hours. Low energy utilization rates are offered during off-peak hours. No peak hour utilization rates are charged on weekends [22]. The pricing mechanism incorporated in this work is illustrated in Table 2. In Queensland, on 26 February 2020, peak and off-peak utilization rates charged were $325.87/MWhr and $151.73/MWhr, respectively [23]. One USD (United States Dollar) is equal to 34.06690740614567 SLRs as of 23 February 2021 [24].

The proposed blockchain-based EDs offer consumers lower energy prices than the conventional grid. Prosumers rate their surplus energy 10 percent lower than rates offered by the traditional grid. Energy exported to SG is 10 percent higher than the normalized rates. Table 3 demonstrates the pricing mechanism exercised on the proposed blockchain platform.

### 2.3. Mutual Energy Contract (MEC)

The nodes of the network agree upon and sign a desired and flexible mutual energy contract to carry out energy transactions without involving a third party. This energy contract across the blockchain platform is referred to as Smart Contract (SC), which is an enforced agreement holding certain rules that need to be followed by every node of the network. SC empowers transparent and trusted energy transactions among nodes of the network and behaves as the controlling entity of a decentralized network [19]. SC is immutable, self-executing, written in the form of code, and is stored on the blockchain [25].

Prosumer mode: Whenever the energy produced by a participant of the network is more than the energy demand, participants announce themselves as prosumers to sell extra, unused energy to grid or neighboring users.

(1) $$\mathrm{Prosumer}\;\mathrm{mode}\approx P_{\mathrm{gen}}{}^{h}>P_{\mathrm{dem}}{}^{h}$$

where $P_{\mathrm{gen}}{}^{h}\;$and $P_{\mathrm{dem}}{}^{h}$ refer to prosumer’s energy generation and demand at hour h, respectively.

Consumer mode: Whenever the energy produced by the participants is not enough to satisfy their energy demand, participants announce themselves as consumers and put an energy request on the network.

(2) $$\mathrm{Consumer}\;\mathrm{mode}\approx P_{\mathrm{gen}}{}^{h}<P_{\mathrm{dem}}{}^{h}$$

Prosumer energy rate: On the blockchain network, prosumers sell energy at a rate 10 percent less than the normalized rates. Consumers buy energy from prosumers on the network at a price offered less than the traditional grid. Prosumers export energy to SG at a rate 10 percent higher than the normalized rates.

(3) $$P_{Pr}{}^{h}\approx \left\{\begin{array}{c} \mathrm{for}\;C,10\%<P_{R}{}^{h}\\ \mathrm{for}\;SG,10\%>P_{R}{}^{h} \end{array}\right.$$

where $P_{Pr}{}^{h}$ is the real-time energy Price (Pr) offered by the Prosumer (P) to Consumer (C) and SG in a blockchain network at hour *h.* $P_{R}{}^{h}$ is the real-time energy price offered by the grid at hour *h.*

Incentives: Solar energy prosumers are rewarded 1 SLR for each MW solar energy produced from the SCF [13].

Energy Transactions: In our proposed model, energy transactions are carried out in four different categories under SC.

(a) Prosumer-to-Consumer (P-C): On the digital blockchain network, prosumers share the information of their energy generation. Consumers reach the prosumer to satisfy load requirements, without relying on the grid.

(4) $$\overset{H}{\underset{h=1}{\sum }}C_{\mathrm{dem}}{}^{h}\leftarrow \overset{H}{\underset{h=1}{\sum }}PES^{h}$$

where $C_{\mathrm{dem}}{}^{h}$ demonstrates the Consumer energy demand at hour *h*, $PES^{h}$ refers to Prosumer Energy Surplus (PES) at hour *h.* Arrow indicates the C energy demand satisfying through PES.

(b) Prosumer-to-Prosumer (P-P): Whenever the prosumers’ energy produced from RE is not enough to meet their load requirements, prosumers import energy from other prosumers in an ED.

(5) $$\overset{H}{\underset{h=1}{\sum }}P_{1,\mathrm{dem}}{}^{h}↔\;\overset{H}{\underset{h=1}{\sum }}P_{2,\mathrm{dem}}{}^{h}$$

where $P_{1,\mathrm{dem}}{}^{h},\;P_{2,\mathrm{dem}}{}^{h}\;$indicate the first and second Prosumer energy demand at hour h, respectively. The arrow specifies mutual energy trade between two prosumers.

(c) Energy District-to-Energy District (ED-ED): An ED fulfills its load requirements from other ED at times of reduced energy generation from RE.

(6) $$\overset{H}{\underset{h=1}{\sum }}ED_{1,\mathrm{dem}}{}^{h}↔\;\overset{H}{\underset{h=1}{\sum }}ED_{2,\mathrm{dem}}{}^{h}$$

where $ED_{1,\mathrm{dem}}{}^{h},\;ED_{2,\mathrm{dem}}{}^{h}$ points out the first ED’s and 2nd ED’s energy demand at hour *h,* respectively.

(d) Energy District-to-Smart Grid (ED-SG): The bi-directional feature and addition of prosumer to SG is beneficial to both SG and prosumer. At times of minimum RE generation, EDs import deficient energy from SG, while at times of maximum RE production, EDs sell surplus and unused energy to SG and are incentivized with SLRs.

(7) $$\overset{H}{\underset{h=1}{\sum }}ED_{\mathrm{dem}}{}^{h}↔\overset{H}{\underset{h=1}{\sum }}SG_{\mathrm{dem}}{}^{h}$$

where $SG_{\mathrm{dem}}{}^{h}$ refers to Smart Grid’s energy demand at hour h.

Constraints: The following constraints are encoded in our implemented SC.

(a) Prosumers and consumers must be connected on the blockchain network.

(8) $$\overset{H}{\underset{h=1}{\sum }}P_{c}{}^{h}=\overset{H}{\underset{h=1}{\sum }}C_{c}{}^{h}=1$$

where $P_{c}{}^{h},\;C_{c}{}^{h}$ in Equation (8) refers to prosumer and consumer connectivity in the blockchain network at hour *h*, respectively.

(9) $$\overset{H}{\underset{h=1}{\sum }}PES^{h}\;\geq \;\overset{H}{\underset{h=1}{\sum }}C_{\mathrm{dem}}{}^{h}$$

(b) Prosumers must have enough surplus energy to satisfy the consumer’s load requirement.
(c) The consumer must not be a defaulter (must have enough SLR to import energy).

(10) $$\overset{H}{\underset{h=1}{\sum }}{\mathrm{Caccount}}^{h}\;\geq \;\overset{H}{\underset{h=1}{\sum }}P_{Pr}{}^{h}$$

where ${\mathrm{Caccount}}^{h}$ demonstrates the consumer’s account status at hour *h* and $P_{Pr}{}^{h}$ refers to th ereal-time energy price offered by prosumer at hour *h*.

(d) Prosumer must sell energy at a reduced rate.

(11) $$\overset{H}{\underset{h=1}{\sum }}P_{Pr}{}^{h}\;\leq \;\overset{H}{\underset{h=1}{\sum }}P_{R}{}^{h}$$

where $P_{R}{}^{h}$ in Equation (11) indicates the real-time energy price encounters by the consumer from the grid.

## 3. Blockchain-Based Robust Optimization Model

The advanced technical appliances contribute to an elevated consumers’ energy consumption profile. The increased energy consumption overturns high energy costs. An effective Demand-Side Management (DSM) technique curtails the consumer’s energy consumption and the energy cost incurred by the consumer is substantial. The bi-directional communication in SG assists for an effective DSM [26]. Moreover, it empowers RE prosumers to import surplus energy to neighboring users and the grid.

In this paper, the implemented pricing mechanism assists in the execution of DSM. Consumers are encouraged to shift their energy consumption pattern towards off-peak periods due to low energy prices offered during off-peak hours. The implementation of the DSM technique results in (a) reduced consumer’s energy cost, (b) increased production of Prosumer Energy Surplus through maximum utilization of RES, and (c) increased Grid Revenue as a result of maximum RE import to the grid. The multi-objective optimization problem is solved using demand-response algorithms. The Genetic Algorithm (GA) Optimization and the Particle Swarm Optimization (PSO) algorithm are implemented in this work.

### 3.1. Prosumer Energy Surplus (PES)

The prosumer energy generated from solar or wind suffers from fluctuations. At times, the energy generated through RE exceeds the prosumer’s energy demand, enabling prosumers to merchandise this excess energy with the neighboring user. This generated energy that outstrips prosumers’ energy demand is referred to as Prosumer Energy Surplus (PES) [27].

(12) $$PES=P_{\mathrm{gen}}-P_{\mathrm{dem}}$$

The system defined in this work maximizes the PES considering certain constraints. The optimization problem of PES is formulated as:

(13) $$\max\sum_{h=1}^{H}\left(P_{\mathrm{gen}}^{h}-P_{\mathrm{dem}}^{h}\right)$$

*Subject to:*

(14) $$0\leq \left(P_{\mathrm{gen}}^{h}\right)\leq \left(P_{\mathrm{gen},\max}^{h}\right)$$

(15) $$0\leq \left(P_{\mathrm{dem}}^{h}\right)\leq \left(P_{\mathrm{dem},\max}^{h}\right)$$

where $P_{\mathrm{gen}}^{h}$ refers to prosumers’ energy generated from wind or solar at hour *h* and $P_{\mathrm{dem}}^{h}$ speaks for prosumers’ actual energy demand at hour *h.* $P_{\mathrm{gen},\max}^{h}$ and $P_{\mathrm{dem},\max}^{h}$ identifies the prosumers’ maximum energy generation and demand, respectively. Equation (13) represents the objective function, and Equations (14) and (15) are inequality constraints of an optimization problem.

### 3.2. Prosumer Energy Cost (PEC)

The prosumer energy demand that exceeds wind- or solar-generated energy compels the prosumer to import this deficient energy from the grid or neighboring users to meet energy requirements. The imported energy cost incurred by the prosumer is referred to as Prosumer Energy Cost (PEC).

(16) $$PEC=P_{\mathrm{dem}}-P_{\mathrm{gen}}$$

The presented model focuses on minimizing PEC through optimization of the prosumer energy demand. The mathematical formulation of the optimization problem for PEC is expressed as:

(17) $$\min\sum_{h=1}^{H}P_{R}^{h}*\left(P_{\mathrm{dem}}^{h}-P_{\mathrm{gen}}^{h}\right)$$

*Subject to:*

(18) $$0\leq \left(P_{\mathrm{gen}}^{h}\right)\leq \left(P_{\mathrm{gen},\max}^{h}\right)$$

(19) $$0\leq \left(P_{\mathrm{dem}}^{h}\right)\leq \left(P_{\mathrm{dem},\max}^{h}\right)$$

where Equation (17) specifies the objective function of the presented optimization problem with Equations (18) and (19) acting as inequality constraints. $P_{R}^{h}$ refers to real-time energy price offered by the grid at hour *h*.

### 3.3. Grid Revenue

The PES exported to the grid results in an incentive for prosumers defined as Grid Revenue (GR), expressed as:

(20) $$GR=(\left(P_{R}*E_{\mathrm{impSG}})+((P_{R}-P_{ɳ})+PES\right))$$

(21) $$\;\max\sum_{h=1}^{H}((P_{R}^{h}*E_{\mathrm{impSG}}^{h})+\left(PES^{h}*(P_{R}^{h}-P_{ɳ}^{h})\right))$$

(22) $$0\leq \left(P_{\mathrm{gen}}^{h}\right)\leq \left(P_{\mathrm{gen},\max}^{h}\right)$$

*Subject to:*

(23) $$0\leq \left(P_{\mathrm{dem}}^{h}\right)\leq \left(P_{\mathrm{dem},\max}^{h}\right)$$

The proposed model emphasizes the maximization of GR. The optimization problem formulation for GR is illustrated in Equations (21)–(23). Equation (21) presents the objective function of the maximization problem while Equations (22) and (23) are inequality constraints of an optimization problem.

$P_{ɳ}^{h}$ is the nominal price proposed to SG on account of prosumer energy export and $E_{\mathrm{impSG}}^{h}$ is the energy imported from SG at hour *h*.

### 3.4. Prosumer Energy Surplus (PES) and Prosumer Energy Cost (PEC)

This research work focuses on the comparative analysis of PES and PEC in one domain subjected to diverse weather fluctuations. The merged mathematical model for the minimization of PEC and maximization of PES is expressed as:

(24) $$\min\sum_{h=1}^{H}P_{R}^{h}*\left(P_{\mathrm{dem}}^{h}-P_{\mathrm{gen}}^{h}\right)-ɳ*\max\sum_{h=1}^{H}\left(P_{\mathrm{gen}}^{h}-P_{\mathrm{dem}}^{h}\right)$$

*Subject to:*

(25) $$0\leq \left(P_{\mathrm{gen}}^{h}\right)\leq \left(P_{\mathrm{gen},\max}^{h}\right)$$

(26) $$0\leq \left(P_{\mathrm{dem}}^{h}\right)\leq \left(P_{\mathrm{dem},\max}^{h}\right)$$

(27) $$0\leq \left({ɳ}^{h}\right)\leq \left(100\right)$$

where *ɳ* is the weighting function.

### 3.5. Optimization Algorithm

This research work uses Particle Swarm Optimization (PSO) and Genetic Algorithm (GA) optimization algorithms for the minimization of PEC and maximization of PES, percentage energy transactions accomplished throughout the year, and GR.

Algorithms 1 and 2 solve the multi-objective optimization problem to carry out the objectives of increased PES, GR, and minimized PEC, respectively.

The layout of GA for the optimization of PEC, PES, and GR is presented as:
**Algorithm 1** Robust GA optimization algorithm for PEC, PES, GR1:   **Initialize**
$P_{R}{}^{h},\;PE_{\mathrm{dem}}{}^{h},\;PE_{\mathrm{gen}}{}^{h}$ 2:   t = 0 3:   **Evaluate**
PES  using (12), PEC using (16) and GR using (20) 4:   **Evaluate** optimization problem for PES, PEC and GR using (13)–(15), (17)–(19), (20)–(23), and (24)–(27) 5:   **If** optimization converges, **then** optimization achieved. 6:                **Else** 7:                t = t + 1 8:                Evaluate next generation. 9:        **End**
10: **End**

Algorithm 2 presents the layout of the PSO technique for PEC, PES, and GR.
**Algorithm 2** Robust PSO algorithm for PEC, PES, GR1:   **Initialize**
$P_{R}{}^{h},\;PE_{\mathrm{dem}}{}^{h},\;PE_{\mathrm{gen}}{}^{h}$ 2:   t = 0 3:   **Evaluate**
 PES using (12), PEC using (16) and GR using (20) 4:   **Evaluate** optimization problem for PES, PEC and GR using (13)–(15), (17)–(19), (20)–(23), and (24)–(27) 5:   **If** optimization converges, **then** optimization achieved. 6:               **Else** 7:               t = t + 1 8:       **End** 9: **End**

### 3.6. Proposed Blockchain Optimization Model

Robust demand-side optimization is considered to be a pivotal element in implementing blockchain-based energy transactions. This work aims at accomplishing maximum energy transactions among participants of the SLR blockchain network throughout the year. The mathematical formulation of the optimization problem is expressed in Equations (28)–(39), with Equations (28)–(31) acting as the objective function of the maximum optimization problem. Equations (32)–(35) and Equations (37)–(39) are inequality constraints of the optimization problem while Equation (36) presents equality constraints of the proposed optimization problem.

(28) $$\max(\overset{H}{\underset{h=1}{\sum }}C_{\mathrm{dem}}{}^{h}\leftarrow \overset{H}{\underset{h=1}{\sum }}PES^{h})$$

(29) $$\max(\overset{H}{\underset{h=1}{\sum }}P_{1,\mathrm{dem}}{}^{h}↔\;\overset{H}{\underset{h=1}{\sum }}P_{2,\mathrm{dem}}{}^{h})$$

(30) $$\max(\overset{H}{\underset{h=1}{\sum }}ED_{1,\mathrm{dem}}{}^{h}↔\;\overset{H}{\underset{h=1}{\sum }}ED_{2,\mathrm{dem}}{}^{h})$$

(31) $$\max(\overset{H}{\underset{h=1}{\sum }}ED_{\mathrm{dem}}{}^{h}↔\overset{H}{\underset{h=1}{\sum }}SG_{\mathrm{dem}}{}^{h})$$

*Subject to:*

(32) $$0\leq \left(C_{\mathrm{dem}}^{h}\right)\leq \left(C_{\mathrm{dem},\max}^{h}\right)$$

(33) $$0\leq \left(P_{1,dem}^{h}\right)\leq \left(P_{1,\mathrm{dem},\max}^{h}\right)$$

(34) $$0\leq \left(P_{2,\mathrm{dem}}^{h}\right)\leq \left(P_{2,\mathrm{dem},\max}^{h}\right)$$

(35) $$0\leq \left(ED_{1,\mathrm{dem}}^{h}\right)\leq \left(ED_{2,\mathrm{dem},\max}^{h}\right)$$

(36) $$\overset{H}{\underset{h=1}{\sum }}P_{c}{}^{h}=\overset{H}{\underset{h=1}{\sum }}C_{c}{}^{h}=1$$

(37) $$\overset{H}{\underset{h=1}{\sum }}PES^{h}\;\geq \;\overset{H}{\underset{h=1}{\sum }}C_{dem}{}^{h}$$

(38) $$\overset{H}{\underset{h=1}{\sum }}{\mathrm{Caccount}}^{h}\;\geq \;\overset{H}{\underset{h=1}{\sum }}P_{Pr}{}^{h}$$

(39) $$\overset{H}{\underset{h=1}{\sum }}P_{Pr}{}^{h}\;\leq \;\overset{H}{\underset{h=1}{\sum }}P_{R}{}^{h}$$

where $C_{\mathrm{dem},\max}^{h}$ is the consumer’s maximum energy demand at hour *h*. $\left(P_{1,\mathrm{dem},\max}^{h}\right)$ represents the first prosumer’s maximum energy demand and $\left(P_{2,\mathrm{dem},\max}^{h}\right)$ illustrates the second prosumer’s maximum energy demand at hour *h*. $\left(ED_{2,\mathrm{dem},\max}^{h}\right)$ represents ED’s maximum demand at hour *h*.

Energy transactions among multiple participants of the proposed model are accomplished utilizing Algorithms 3 and 4.

The layout of the energy transactions carried out among multiple prosumers, consumers, EDs, and SG is portrayed through the following algorithms:
**Algorithm 3** Blockchain-based P-C energy transaction algorithm1:   **Initialize**
$PES^{h},\;C_{dem}^{h}$, $P_{Pr}^{h}$, 2:   Gather prosumer’s and consumer’s active status on the blockchain network 3:   Gather consumer’s account details. 4:   **Accomplish** energy transaction between Prosumer to the Consumer using (28) subject to the constraints in (32)–(39) 5:   **If** the transaction successful 6:       Energy transferred to the consumer, SLRs transferred to prosumer. 7:       **Else** 8:          No energy transacted, shift to another prosumer. 9:          Repeat step 1–10 for another energy transaction 10: **End** 11: **End****Algorithm 4** Blockchain-based P-P, ED-ED, and ED-SG energy transactions algorithm1:   **Initialize**
$P_{1}ES^{h},\;P_{2}ES^{h},\;P_{1}{}_{dem}^{h}$, $P_{2}{}_{\mathrm{dem}}^{h},\;P_{Pr}^{h}$, 2:   Gather prosumer1 and prosumer2 active status on the blockchain network. 3:   Gather prosumer1 and prosumer2 account details. 4:   Decide prosumer and consumer from prosumer1 and prosumer2. 5:   **Accomplish** bi-directional energy transaction between Prosumer to Prosumer, Energy District to Energy District and Energy District to Smart Grid using (29)–(31) subject to the constraints in (32)–(39) 6:   **If** the transaction successful 7:        Energy transferred to the consumer, SLRs transferred to prosumer. 8:     **Else** 9:        No energy transacted, shift to another prosumer. 10:         Repeat step 1–10 for another energy transaction 11: **End** 12: **End**

## 4. Performance Evaluation

This section arrays the implementation of SLR blockchain-based METM. An in-depth analysis accompanied by the detailed results of the proposed model is incorporated in this section.

### 4.1. Data Analysis

The proposed model in this article is evaluated regulating Australian-based case studies. The energy generation and consumption data profiles of one year from Queensland (Australia) are considered. For the simulation of each day, energy data of 24 h are considered. We have considered independent solar energy producers, and their energy profiles are taken from Queensland Live Solar Outputs [28]. Two onshore wind farms, Cooper Gap Wind Farm (COOPGWF1) and Mount Emerald Wind Farm (MEWF1), with the generating capacity of 452 and 180 MW, respectively, are considered. Their hourly energy generation data are extracted from Aneroid Energy [29]. The energy demand for Queensland and grid generation data are extracted from AEMO [30].

### 4.2. Seasonal Variations

In our presented case study, blockchain-based energy transactions and DSM are analyzed under the influence of four seasons i.e., Spring, Summer, Autumn, and Winter seasons.

#### 4.2.1. Spring Season

**Figure 2:**
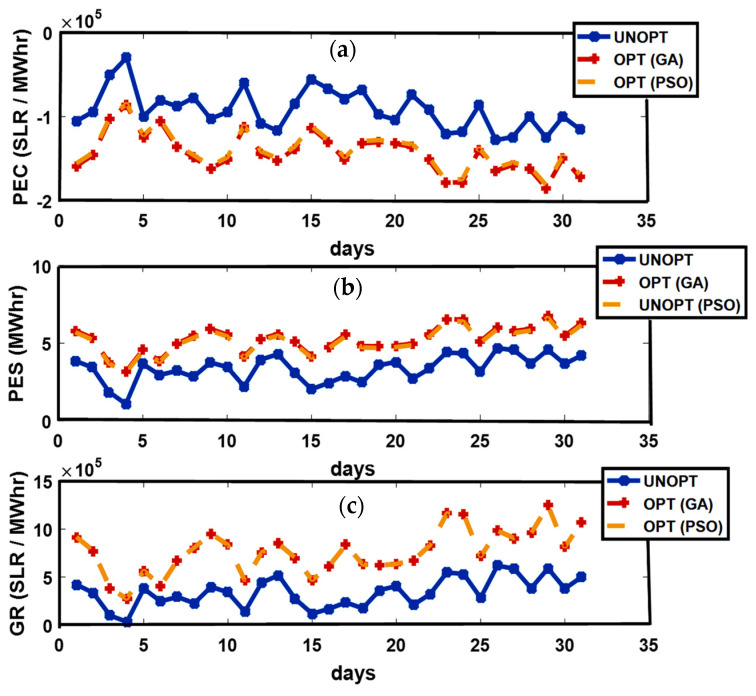
(**a**): Prosumer Energy Cost (PEC); (**b**): Prosumer Energy Surplus (PES); (**c**): Grid Revenue (GR).

Australian seasons come about at times opposite to the northern hemisphere. The Spring season is observed from September to November [31]. In Queensland, September and October are the months experiencing the best solar performance with 7.25 and 6.28 peak sun h/day [32]. These months are considered as windier months of the year with an average wind speed exceeding 4.2 m/s [33]. Prosumers have enough energy generation, and export surplus energy to neighboring users and the grid. Increased export and decreased import from the grid overturns reduced PEC. Figure 2 shows the unoptimized and optimized PEC, PES, and GR. ED1 and the month of October are considered for graphical analysis. The 9th, 14th, 23rd, 24th, and 29th of October have the maximum solar irradiance resulting in escalated solar energy generation and minimum PEC; while on the 4th, 10th, and 15th of October, minimum solar energy is produced, compelling the prosumer to import energy from the grid, resulting in an increased PEC. The figure demonstrates that the maximum solar energy is produced on the 9th, 14th, 23rd, 24th, and 29th of October due to increased solar radiation resulting in high PES. The minimum solar energy is generated on the 4th, 10th, and 15th of October leading to decreased PES. The maximum generation of PES on the 9th, 14th, 23rd, 24th, and 29th of October permits the prosumer to sell extra energy to the grid, paving the way for an escalated GR; while the minimum PES generation on the 4th, 10th, and 15th of October results in decreased GR.

**Figure 3:**
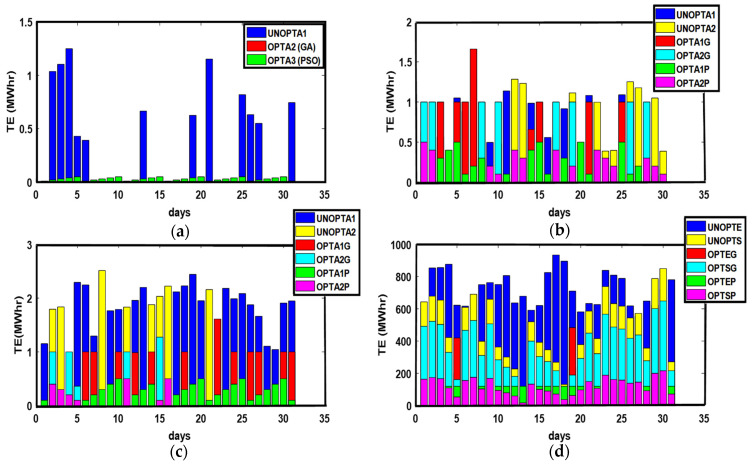
(**a**) P-C, (**b**) P-P, (**c**) ED-ED, and (**d**) ED-SG Energy Transactions. (**a**) TE: Transacted Energy, UNOPTA1: Unoptimized Consumer Energy, OPTA2 (GA): GA Optimized consumer energy, OPTA3 (PSO): PSO consumer energy; (**b**) UNOPTA1: Prosumer 1 Unoptimized transacted energy, UNOPTA2: Prosumer 2 Unoptimized transacted energy, OPTA1G: Prosumer 1 GA Optimized transacted energy, OPTA2G: Prosumer 2 GA Optimized transacted energy, OPTA1P: Prosumer 1 PSO transacted energy, OPTA2P: Prosumer 2 PSO transacted energy; (**c**) UNOPTA1: ED1 Unoptimized transacted energy, UNOPTA2: ED2 Unoptimized transacted energy, OPTA1G: ED1 GA Optimized transacted energy, OPTA2G: ED2 GA Optimized transacted energy, OPTA1P: ED1 PSO transacted energy, OPTA2P: ED2 PSO transacted energy; (**d**) UNOPTE: ED Unoptimized transacted energy, UNOPTS: SG Unoptimized transacted energy, OPTEG: ED GA Optimized transacted energy, OPTSG: SG GA Optimized transacted energy, OPTEP: ED PSO transacted energy, OPTSP: SG PSO transacted energy.

The energy transactions between P-C, P-P, ED-ED, and ED-SG are presented in Figure 3. The optimized results outweigh the unoptimized results. GA and PSO maximize the PES and GR and minimize PEC. Similarly, more optimized energy transactions are performed than unoptimized transactions due to the maximization of PES. Due to the availability of surplus energy, ED imports surplus unused energy to SG, as shown in Figure 3.

#### 4.2.2. Summer Season

**Figure 4:**
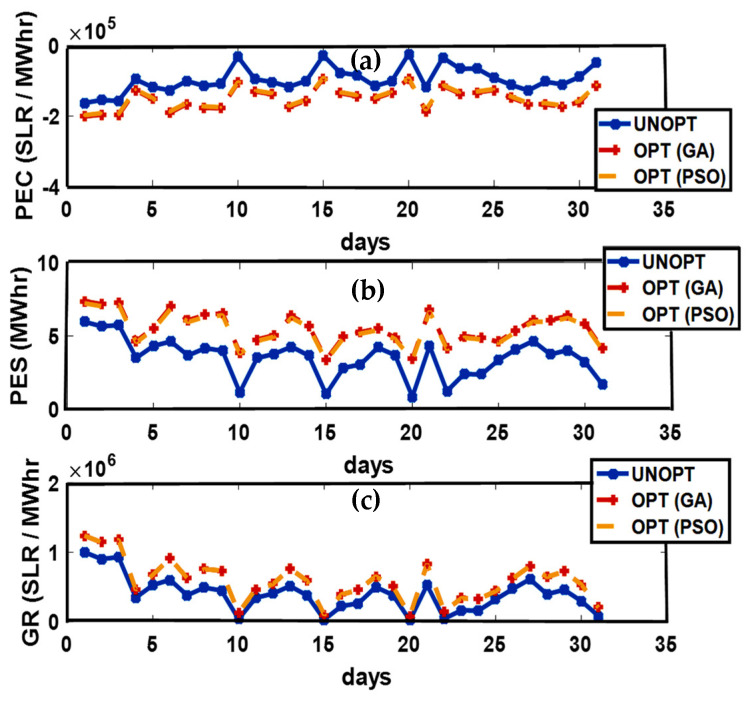
(**a**) Prosumer Energy Cost (PEC), (**b**) Prosumer Energy Surplus (PES), (**c**) Grid Revenue (GR).

**Figure 5:**
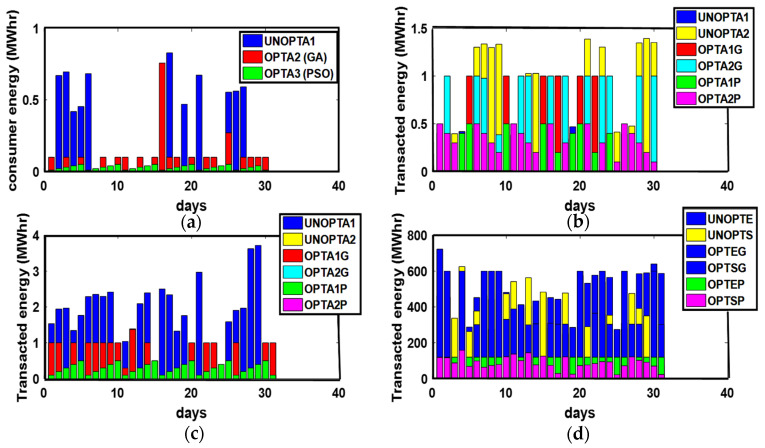
(**a**) P-C, (**b**) P-P, (**c**) ED-ED, and (**d**) ED-SG Energy Transactions. (**a**) UNOPTA1: Unoptimized Consumer Energy, OPTA2 (GA): GA Optimized consumer energy, OPTA3 (PSO): PSO consumer energy; (**b**) UNOPTA1: Prosumer 1 Unoptimized transacted energy, UNOPTA2: Prosumer 2 Unoptimized transacted energy, OPTA1G: Prosumer 1 GA Optimized transacted energy, OPTA2G: Prosumer 2 GA Optimized transacted energy, OPTA1P: Prosumer 1 PSO transacted energy, OPTA2P: Prosumer 2 PSO transacted energy; (**c**) UNOPTA1: ED1 Unoptimized transacted energy, UNOPTA2: ED2 Unoptimized transacted energy, OPTA1G: ED1 GA Optimized transacted energy, OPTA2G: ED2 GA Optimized transacted energy, OPTA1P: ED1 PSO transacted energy, OPTA2P: ED2 PSO transacted energy; (**d**) ED-SG Energy Transaction, UNOPTE: ED Unoptimized transacted energy, UNOPTS: SG Unoptimized transacted energy, OPTEG: ED GA Optimized transacted energy, OPTSG: SG GA Optimized transacted energy, OPTEP: ED PSO transacted energy, OPTSP: SG PSO transacted energy.

In Australia, the Summer season is observed from December to February. Average solar performance during the summer season is recorded as 6.22 peak sun h/day. The months in the Summer season are considered as the windier part of the year with an average wind speed of 4.2 m/s. February 26th is recorded as the windiest day of the year with an average wind speed of 4.5 m/s [33]. Excellent solar irradiance and wind speed contribute to an increased PES and minimized PEC. Unoptimized and optimized PEC, PES, and GR are presented in Figure 4. Maximum PEC is observed on 10th, 15th, 20th, 23rd, and 31st of January while the 1st, 5th, 12th, 18th, 21st, and 30th of January flag the minimum PEC. Due to high solar irradiance observed on the 1st, 5th, 12th, 18th, 21st, and 30th of January, the maximum energy surplus is produced. On the 10th, 15th, 20th, 23rd, and 31st of January, the minimum energy surplus is produced. The maximum GR is marked on the 3rd of January while minimum GR is observed on the 10th, 15th, and 20th of January due to the minimum energy production. The energy transactions between P-C, P-P, ED-ED, and ED-SG are presented in Figure 5.

Due to the availability of energy surplus, enough energy transactions are performed between P-C, P-P, and ED-ED throughout the month. Increased PES permits prosumer to export energy to the grid as illustrated in Figure 5.

#### 4.2.3. Autumn Season

**Figure 6:**
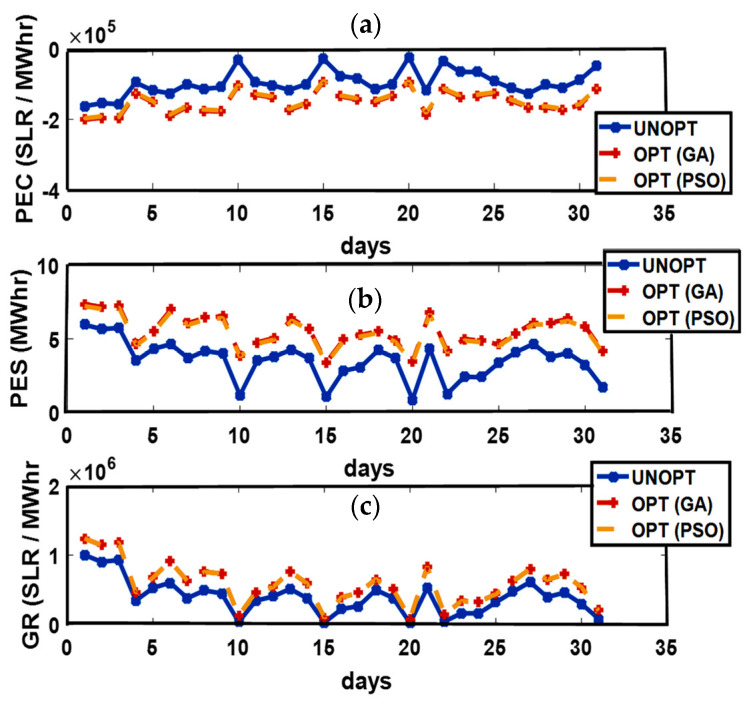
(**a**) Prosumer Energy Cost (PEC), (**b**) Prosumer Energy Surplus (PES), (**c**) Grid Revenue (GR).

**Figure 7:**
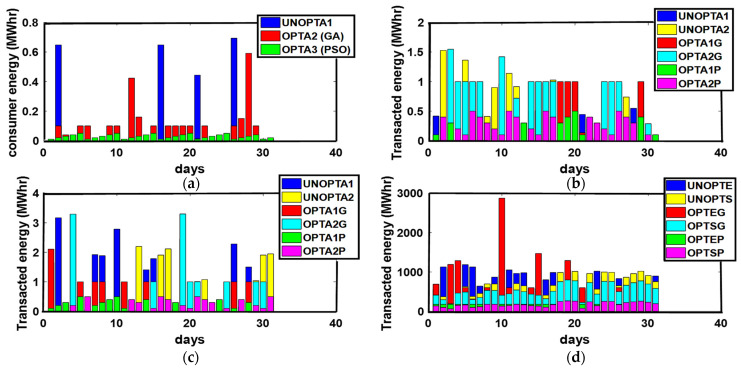
(**a**) P-C, (b) P-P, (**c**) ED-ED, and (**d**) ED-SG Energy Transactions. (**a**) UNOPTA1: Unoptimized Consumer Energy, OPTA2 (GA): GA Optimized consumer energy, OPTA3 (PSO): PSO consumer energy; (**b**) UNOPTA1: Prosumer 1 Unoptimized transacted energy, UNOPTA2: Prosumer 2 Unoptimized transacted energy, OPTA1G: Prosumer 1 GA Optimized transacted energy, OPTA2G: Prosumer 2 GA Optimized transacted energy, OPTA1P: Prosumer 1 PSO transacted energy, OPTA2P: Prosumer2 PSO transacted energy; (**c**) UNOPTA1: ED1 Unoptimized transacted energy, UNOPTA2: ED2 Unoptimized transacted energy, OPTA1G: ED1 GA Optimized transacted energy, OPTA2G: ED2 GA Optimized transacted energy, OPTA1P: ED1 PSO transacted energy, OPTA2P: ED2 PSO transacted energy; (**d**) UNOPTE: ED Unoptimized transacted energy, UNOPTS: SG Unoptimized transacted energy, OPTEG: ED GA Optimized transacted energy, OPTSG: SG GA Optimized transacted energy, OPTEP: ED PSO transacted energy, OPTSP: SG PSO transacted energy.

The Autumn season is observed in Queensland, Australia in March, April, and May, where 4.39 peak sun h/day is recorded as the average sun performance during the Autumn season with an average wind speed of 3.5 m/s. The maximum surplus energy generation on the 3rd and 16th of March results in minimized PEC as depicted from Figure 6. The increased PES on the 3rd, 11th, 16th, 20th, and 29th of March permits ED to export energy to the grid, contributing to escalated GR. Figure 7 presents energy transactions performed between P-C, P-P, ED-ED, and ED-SG, respectively.

#### 4.2.4. Winter Season

**Figure 8:**
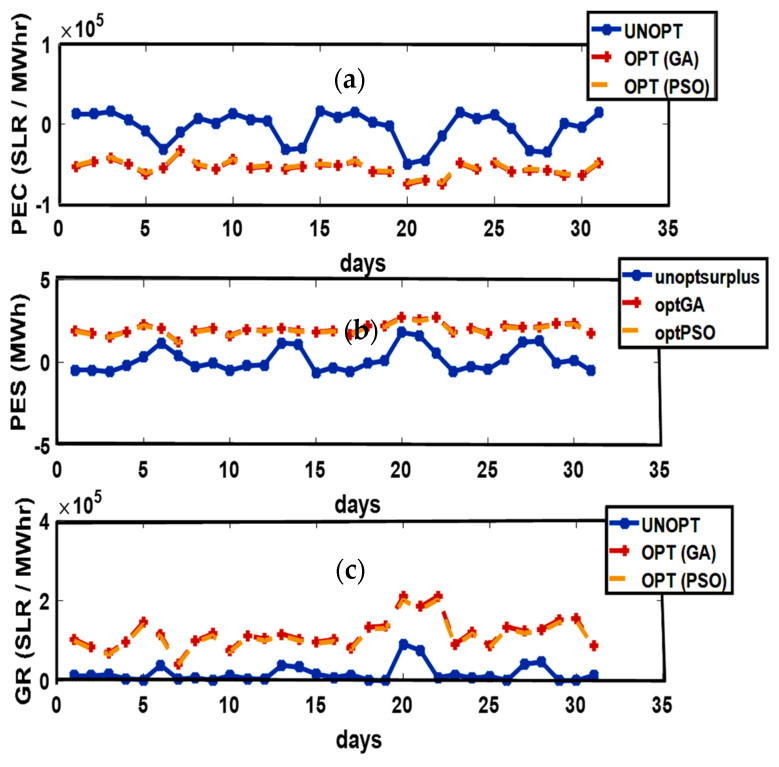
(**a**) Prosumer Energy Cost (PEC), (**b**) Prosumer Energy Surplus (PES), (**c**) Grid Revenue (GR).

**Figure 9:**
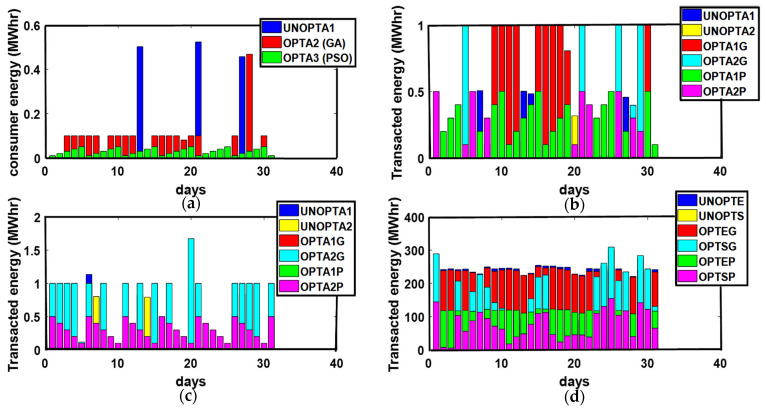
(**a**) P-C, (**b**) P-P, (**c**) ED-ED, and (**d**) ED-SG Energy Transactions. (**a**) UNOPTA1: Unoptimized Consumer Energy, OPTA2 (GA): GA Optimized consumer energy, OPTA3 (PSO): PSO consumer energy; (**b**) UNOPTA1: Prosumer 1 Unoptimized transacted energy, UNOPTA2: Prosumer 2 Unoptimized transacted energy, OPTA1G: Prosumer 1 GA Optimized transacted energy, OPTA2G: Prosumer 2 GA Optimized transacted energy, OPTA1P: Prosumer 1 PSO transacted energy, OPTA2P: Prosumer 2 PSO transacted energy; (**c**) ED-ED Energy Transaction, UNOPTA1: ED1 Unoptimized transacted energy, UNOPTA2: ED2 Unoptimized transacted energy, OPTA1G: ED1 GA Optimized transacted energy, OPTA2G: ED2 GA Optimized transacted energy, OPTA1P: ED1 PSO transacted energy, OPTA2P: ED2 PSO transacted energy; (**d**) ED-SG Energy Transaction, UNOPTE: ED Unoptimized transacted energy, UNOPTS: SG Unoptimized transacted energy, OPTEG: ED GA Optimized transacted energy, OPTSG: SG GA Optimized transacted energy, OPTEP: ED PSO transacted energy, OPTSP: SG PSO transacted energy.

The Winter season is observed in Queensland, Australia from June to August. June has the worst average solar performance with 5.36 peak sun h/day. The average wind speed in Queensland from June to August is 3.8 m/s. The optimized and unoptimized PEC, PES, and GR are presented in Figure 8. The 4th, 10th, 15th, 24th, and 31st of July have the maximum while the 6th, 13th, 14th, 20th, 26th, and 27th of July have the minimum PEC. The maximum PES is observed on the 6th, 13th, 14th, 20th, 26th, and 27th of July. The 4th, 10th, 15th, 24th, and 31st of July exhibits low PES leading prosumer towards grid energy import and a reduced PEC. Days with low PES (4th, 10th, 15th, 24th, and 31st of July) result in decreased GR while the maximum GR is observed on the days of high PES. Figure 9 demonstrates the number of unoptimized and optimized energy transactions performed between P-C, P-P, ED-ED, and ED-SG throughout the month. Due to the minimum wind speed and solar radiance, minimal energy transactions are accomplished throughout the month. GA maximizes the number of energy transactions more so than PSO as depicted in Figure 9.

#### 4.2.5. Comparative Analysis of Genetic Algorithm (GA) Optimization and Particle Swarm Optimization (PSO)

Optimization of PEC, PES, and GR performed through the GA and PSO algorithms is comparatively analyzed in this work. Optimized results achieved through GA outperform the PSO results. The maximum reduction of PEC is accomplished using GA in all four seasons compared to PSO. Similarly, GA assists in the maximization of PES and GR more effectively than PSO.

Table 4 summarizes the average results of GA optimization and PSO for three EDs. The table shows average change observed between unoptimized and optimized results of GA is more than the average change of PSO results and signifies the better performance of GA in this work.

#### 4.2.6. SolarCoin (SLR) Blockchain Implementation

**Figure 10:**
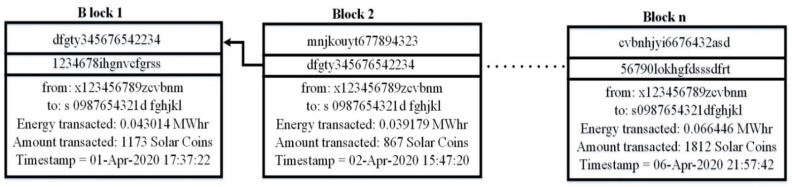
Blockchain transaction.

Figure 10 demonstrates energy transactions performed between a prosumer and a consumer on the SLR blockchain. The record of the transaction exhibits an encrypted hash of the block, the address of prosumer and consumer, the time of the transaction, and the amount of energy /SLR transacted. A consumer with the address “s0987654321dfghjkl” requests 0.043014 MW of energy on the network. The system searches for a prosumer on the network owning 0.043014 MW surplus energy. The system connects the consumer with the prosumer with the address “x123456789zcvbnm”. SC executes and implements the encoded constraints. On successful implementation of constraints, the prosumer sells the required energy to the consumer and the consumer pays the prosumer with 1172 SLRs.

### 4.3. Prosumer Energy Cost (PEC) and Prosumer Energy Surplus (PES)

**Figure 11:**
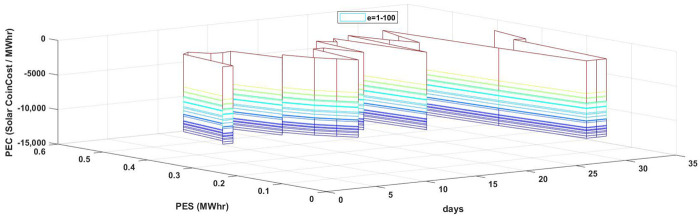
PES and PEC comparison.

Figure 11 signifies the impact of the variation of PES on PEC. An increase in the PES on account of the increased peak sun h/day and increased wind speed eliminates the need for energy import from the grid, resulting in a decreased PEC as depicted in Figure 11. For each MWhr rise in PES, PEC subsequently decreases.

### 4.4. Critical Analysis

The comparative tabular analysis of unoptimized and optimized GA DSM is scrutinized in this sub-section. Aggregate energy transactions accomplished in all four seasons are extensively analyzed.

#### 4.4.1. Comparative Analysis of Demand Management

Table 5 presents the comparative statistics of DSM performed on the proposed SLR blockchain-based METM. Unoptimized and GA-optimized results for ED1, ED2, and ED3 enveloping PEC, PES, and GR are manifested in this table. The average change observed between unoptimized and optimized results of the three EDs is analyzed. The table shows GA optimized results surpass the unoptimized results. PEC is effectively minimized throughout the year as a result of optimization of EDs’ demand. Similarly, energy surplus is maximized empowering prosumers to satisfy their load requirements and import energy to SG, resulting in an increased GR for prosumers.

#### 4.4.2. Smart Contract Validation

Extensive variation in solar radiation and wind speed throughout the year influence the aggregated RE generation engendering enough or reduced energy transactions. The averaged percentage of unoptimized, GA-optimized, and PSO-based energy transactions accomplished in each month are summarized in Table 6. The table shows that the percentage of optimized transactions is more than the percentage of unoptimized energy transactions. In addition, the percentage change of GA optimized energy transactions is greater than the average change observed in PSO.

## 5. Conclusions and Future Work

A central authority supervising energy and financial transactions in an energy sector experiences a substantial computational burden. Stakeholders are being charged for each transaction made by a central authority and the privacy of their transactions’ profile is greatly at threat. The trading approach in a decentralized fashion bypassing central authority is greatly in need. Blockchain technology underpins peer-to-peer energy transactions and eliminates the issues associated with the central authority. SLR blockchain-based METM is proposed to permit a decentralized energy trading concept. Two Solar EDs and a Wind ED incorporating N-SEPs, N-WEPs, and N-consumers are deployed in the proposed model. Energy transactions are carried out between P-C, P-P, ED-ED, and ED-SG. SLRs are exchanged among the users as a result of energy trading. The demand-side optimization problem is formulated using GA and PSO for the maximization of PES and GR and minimization of PEC. The performance of the SLR blockchain-based METM is evaluated in all four seasons. The unoptimized, GA-optimized, and PSO results of four seasons are critically and comparatively analyzed. Maximum energy surplus is generated in the Summer and Spring (52 percent) seasons on account of the high wind speed and enough solar radiations. Seventy-one percent of GR is earned in the Summer and Spring seasons. Similarly, the minimum PEC is observed in the Spring and Summer (53 percent) seasons compared to the Autumn and Winter seasons. The GA optimized results outperform the PSO results. GA maximizes PES on average by 29 percent compared to PSO (28 percent). The uncertainty confronted by the RE generation affects the average energy transactions carried out in each season. Thirty-nine percent of energy transactions are accomplished between P-C in the Spring season and 35 percent in the Summer season compared to Autumn (13 percent) and Winter (10 percent) season. Similarly, GA effectively maximizes the percentage of energy transactions accomplished as compared to PSO. A 62 percent average increase in energy transactions carried out between P-C by GA optimization is observed in the Summer season compared to that carried out by PSO (60 percent).

The scalability of our proposed model incorporating multiple energy transactions among numerous EDs and SG at one time is a possible future direction of this work. Furthermore, issues coupled with the scalability of blockchain technology need to be addressed in the future. Speed and security are the major directions of improvement in a scaled blockchain network.

## Figures and Tables

**Table 1:** Literature Review

Ref.	DSM	BC	SLR-BC	SLRD	O-PEC	O-PES	O-GR	O-ET
**[2]**	🗴	🗸	🗴	🗴	🗴	🗴	🗴	🗴
**[3]**	🗴	🗸	🗴	🗴	🗴	🗴	🗴	🗴
**[5]**	🗴	🗸	🗴	🗴	🗴	🗴	🗴	🗴
**[11]**	🗴	🗸	🗴	🗴	🗴	🗴	🗴	🗴
**[12]**	🗸	🗸	🗴	🗴	🗸	🗴	🗴	🗸
**[13]**	🗴	🗸	🗴	🗴	🗴	🗴	🗴	🗴
**[14]**	🗸	🗸	🗴	🗴	🗸	🗴	🗴	🗸
**[15]**	🗴	🗸	🗴	🗴	🗴	🗴	🗴	🗴
**[16]**	🗸	🗸	🗴	🗴	🗸	🗴	🗴	🗸
**[17]**	🗴	🗸	🗴	🗴	🗴	🗴	🗴	🗴
**[18]**	🗴	🗸	🗴	🗴	🗴	🗴	🗴	🗴
**TW**	**🗸**	**🗸**	**🗸**	**🗸**	**🗸**	**🗸**	**🗸**	**🗸**

Abbreviations: DSM: Demand-side Management; BC: Blockchain; SLR-BC: SolarCoin Blockchain; SLRD: SolarCoin Disbursement; O-PEC: Optimized Prosumer Energy Cost; O-PES: Optimized Prosumer Energy Surplus; O-GR; Optimized Grid Revenue; O-ET: Optimized Energy Transactions; TW: This Work.

**Table 2:** The implemented energy price mechanism.

Time of Utilization	Amount of Energy Consumed (MWhr)	Energy Price in Dollars ($/MWhr)	Energy Price in SLRs (SLR/MWhr)
Off-peak	0.2	151.73 × 0.2 = 30.3	30.3 × 94.0733 = 2854
On-peak	0.2	325.87 × 0.2 = 65.1	65.1 × 94.0733 = 6131

**Table 3:** Implemented price mechanism in a blockchain.

Time of Utilization	Amount of Energy Consumed (MWhr)	Energy Price in Dollars ($/MWhr, 10 Percent Discounted Rates)	Energy Price in SLRs (SLR/MWhr)
Off-peak	0.2	27.2	2556
On-peak	0.2	58.5	5517

**Table 4:** Comparative analysis of GA and PSO optimized results.

		GA optimization Results			PSO Results		
EDs	Month, Year	ΔPEC (×10^5^)	ΔPES	ΔGR (×10^5^)	ΔPEC (×10^5^)	ΔPES	ΔGR (×10^5^)
	July, 19	2.76	1.9217	0.918	2.75	1.8840	0.877
	Aug, 19	0.24	0.8815	0.9407	0.228	0.836	0.8773
	Sep, 19	0.86	2.838	4.3440	0.242	2.757	4.157
**ED1**	Oct, 19	0.53	1.9505	4.9613	0.501	1.836	4.9613
	Nov, 19	0.531	1.949	2.9284	0.512	1.878	2.783
	Dec, 19	0.302	1.109	2.3639	0.279	1.025	2.158
	Jan, 20	0.39	1.407	2.369	0.3446	1.263	2.369
	Feb, 20	0.295	1.042	2.2345	0.284	1.042	2.051
	Mar, 20	0.325	1.237	1.1018	0.314	1.151	1.025
	April, 20	0.529	1.976	3.1470	0.5188	1.902	2.987
	July, 19	0.487	1.786	2.6009	0.46881	1.718	2.468
	Aug, 19	0.605	2.218	3.4539	0.5845	2.142	3.389
**ED2**	Sep, 19	0.7293	2.674	5.7227	0.70208	2.573	5.4344
	Oct, 19	0.6060	2.2218	3.9258	0.5834	2.138	3.7288
	Nov, 19	0.5389	2.2251	1.3293	0.5883	2.1815	1.336
	Dec, 19	0.58864	2.0396	1.1556	0.5271	1.9996	1.1097
	Jan, 20	0.4294	1.574	1.1538	0.4182	1.5331	1.106
	Feb, 20	0.4118	1.5095	1.7659	0.3983	1.46	1.435
	Mar, 20	0.8454	3.0994	5.0045	0.822	3.0129	4.791
	April, 20	0.64577	2.3672	2.0054	0.6311	2.313	5.235
	July, 19	29.535	108.2	6839.2	28.64	105	6537
	Aug, 19	35.674	130.7	9327.9	34.649	127	8924
**ED3**	Sep, 19	33.122	121.4	11,238	31.92	117	10,685
	Oct, 19	34.794	127.5	12,047	33.55	123	11,461
	Nov, 19	41.191	150.9	15,201	39.832	146	14,495
	Dec, 19	40.268	147.6	13,396	39.013	143	12,792
	Jan, 20	46.878	171.82	15,548	45.561	167	14,883
	Feb, 20	34.109	125.02	10,063	33.012	121	9602
	Mar, 20	40.216	147.4	21,058	38.468	141	19,888
	April, 20	35.062	128.51	29,995	33.284	122	18,234

**Abbreviations:** ∆PEC: Average change between optimized and unoptimized PEC; ∆PES: Average change between optimized and unoptimized PES; ∆GR: Average change between optimized and unoptimized GR.

**Table 5:** Comparative analysis of unoptimized and GA optimized results.

EDs	Month, Year	PEC (un-opt) (×10^5^)	PEC (opt)(×10^5^)	ΔPEC (×10^5^)	PES (un-opt)	PES (opt)	ΔPES	GR (un-opt) (×10^5^)	GR (opt) (×10^5^)	ΔGR (×10^5^)
	July, 19	1.94	−4.70	2.76	0	1.9217	1.9217	0.12386	1.0412	0.9182
	Aug, 19	−0.393	−0.633	0.24	1.4412	2.3227	0.8815	0.58619	1.5226	0.9407
	Sep, 19	−0.35	−1.21	0.86	1.2924	4.1307	2.838	0.47139	4.8154	4.3440
**ED1**	Oct, 19	−1.05	−1.58	0.53	3.8686	5.8191	1.9505	4.2237	9.185	4.9613
	Nov, 19	−0.46	−0.991	0.531	1.6862	3.6359	1.949	0.80243	3.7309	2.9284
	Dec, 19	−0.878	−1.18	0.302	3.2188	4.3286	1.109	2.9240	5.2879	2.3639
	Jan, 20	−1.62	−2.01	0.39	5.9638	7.3715	1.407	10.038	12.407	2.369
	Feb, 20	−0.80913	−1.104	0.295	2.9658	4.0082	1.042	2.4824	4.7169	2.2345
	Mar, 20	−0.8605	−1.186	0.325	3.1544	4.3921	1.237	2.8082	3.9100	1.1018
	April, 20	−0.499	−1.0289	0.529	1.8320	3.8088	1.976	0.94719	4.0942	3.1470
	July, 19	−0.46	−0.947	0.487	1.6861	3.4726	1.786	0.80233	3.4033	2.6009
	Aug, 19	−0.4499	−1.055	0.605	1.6492	3.8676	2.218	0.76760	4.2215	3.4539
**ED2**	Sep, 19	−0.66962	−1.399	0.7293	2.4544	5.1285	2.674	1.7001	7.4228	5.7227
	Oct, 19	−0.55098	−1.157	0.6060	2.0195	4.2413	2.2218	1.1510	5.0768	3.9258
	Nov, 19	0.06806	−0.6070	0.5389	0	2.2251	2.2251	0.068	1.3973	1.3293
	Dec, 19	0.01836	−0.556	0.58864	0	2.0396	2.0396	0.01836	1.1740	1.1556
	Jan, 20	−0.1396	−0.569	0.4294	0.5117	2.0857	1.574	0.07389	1.2277	1.1538
	Feb, 20	−0.2762	−0.688	0.4118	1.0124	2.5219	1.5095	0.02892	1.7949	1.7659
	Mar, 20	−0.3576	−1.203	0.8454	1.3110	4.4104	3.0994	0.48506	5.4896	5.0045
	April, 20	−0.10343	−0.7492	0.64577	0.3791	2.7463	2.3672	0.04056	2.0460	2.0054
	July, 19	−15.769	−45.304	29.535	57.8000	166.05	108.2	942.85	7782.1	6839.2
	Aug, 19	−16.642	−52.316	35.674	61	191.76	130.7	1050.1	10,378	9327.9
**ED3**	Sep, 19	−28.183	−61.305	33.122	103.300	224.70	121.4	3011.5	14,250	11,238
	Oct, 19	−28.264	−63.058	34.794	103.600	231.13	127.5	3029.1	15,077	12,047
	Nov, 19	−28.073	−69.264	41.191	102.900	253.87	150.9	2988.3	18,190	15,201
	Dec, 19	−23.736	−64.004	40.268	87	234.60	147.6	2136.1	15,533	13,396
	Jan, 20	−20.298	−67.176	46.878	74.4000	246.22	171.82	1562.2	17,111	15,548
	Feb, 20	−21.853	−55.962	34.109	80.1000	205.12	125.02	1810.7	11,874	10,063
	Mar, 20	−48.944	−89.160	40.216	179.400	326.80	147.4	9083.1	30,142	21,058
	April, 20	−55.601	−90.663	35.062	203.800	332.31	128.51	11,722	31,167	29,995

**Table 6:** Percentage energy transactions.

Nodes	Month, Year	% ET (un-opt)	% ET (opt-GA)	Δ% ET (GA)	% ET (opt-PSO)	Δ% ET (PSO)
	July, 19	10	100	90	100	90
	Aug, 19	35	100	65	100	65
	Sep, 19	7	97	90	96	89
	Oct, 19	39	94	55	93	54
**P-C**	Nov, 19	40	100	60	100	60
	Dec, 19	42	100	58	99	57
	Jan, 20	35	97	62	97	62
	Feb, 20	14	100	86	98	84
	Mar, 20	13	100	87	97	84
	April, 20	19	100	81	99	80
	July, 19	11	43	32	42	31
	Aug, 19	32	50	18	50	18
	Sep, 19	29	48	19	48	19
	Oct, 19	45	48	3	48	3
**P-P**	Nov, 19	47	48	1	46	1
	Dec, 19	47	48	1	47	0
	Jan, 20	45	48	3	48	3
	Feb, 20	72	90	18	89	17
	Mar, 20	31	43	12	41	10
	April, 20	30	42	12	40	10
	July, 19	10	48	38	46	36
	Aug, 2019	20	48	28	47	27
	Sep, 19	35	50	15	49	14
	Oct, 19	48	50	2	50	2
**ED-ED**	Nov, 19	48	52	4	51	3
	Dec, 19	52	70	18	69	17
	Jan, 20	37	50	13	48	11
	Feb, 20	50	46	4	46	4
	Mar, 20	42	48	6	46	4
	April, 20	39	40	1	40	1

## Data Availability

This study did any report any data.

## Nomenclature

**Acronyms**	**Definitions**
C	Consumer
DRP	Demand Response Program
DSM	Demand-Side Management
EDs	Energy Districts
EM	Energy Management
GA	Genetic Algorithm
GR	Grid Revenue
MEC	Mutual Energy Contract
METM	Mutual Energy Trade Model
P	Prosumer
PEC	Prosumer Energy Cost
PES	Prosumer Energy Surplus
PSO	Particle Swarm Optimization
P2P	Peer-to-peer
RE	Renewable Energy
SC	Smart Contract
SEPs	Solar Energy Prosumers
SG	Smart Grid
SLRs	Solar Coins
WEPs	Wind Energy Prosumers
